# 

**Published:** 1913-02-08

**Authors:** 


### The Metropolitan Hospital Sunday Fund.

### Date of the Special General Meeting.

The following have been appointed the Distribution Committee of the Metropolitan Hospital Sunday Fund for the year—viz.: Prebendary Anderson, Sir Savile Crossley, Sir William Church, Mr. F. A. Bevan, Mr. Robert Grey, Mr. G. H. Makins, C.B., Mr. Parker Young, Sir Marcus Samuel, and Mr. Adrian Pollock (City Chamberlain). The honorary secretaries chosen were Sir R. B. Martin and Mr. John Henry Buxton, the latter replacing the late Mr. J. S. Gilliat. Messrs. W. H. Pannell and Co. have been re-appointed auditors.

At the meeting of the Council, at which this committee was appointed, the following resolution was brought forward on the motion of the Rev. W. Hardy Harwood, seconded by Sir Henry Burdett, and it was resolved: "That the laws of the constitution of the Fund be referred to a small special committee to bring up rules or by-laws regulating the procedure which shall in future govern the proceedings at the meetings of constituents, and that these by-laws, when approved by the Council, shall be laid before a special meeting of the constituents to be held at the earliest possible date."

A special general meeting of the constituents of the Fund has been summoned by the Lord Mayor for Monday, February 10, at 3.30, at the Mansion House, to consider the three motions by the Rev. L. S. Lewis, the text of which was published in The Hospital of December 28, page 360.

### PRIVATE ARRANGEMENTS IN MIDDLESEX.

At a meeting of the Middlesex Insurance Committee on Monday the General Purposes Sub-Committee recommended that in no circumstances should any insured persons be allowed to make private arrangements for medical benefit for the period up to April 14 next with a medical practitioner who had withdrawn from his contract, and that only in exceptional circumstances should persons be allowed to make their own arrangements for medical benefit, each case to be dealt with on its merits.

Mr. W. Davies said it was an absolute scandal that they were going to try to force people willy-nilly to go to some doctor on the panel.

The Chairman (Mr. Glyn-Jones, M.P.) replied that in certain areas the applications for "contracting out" would not have numbered forty but for the direct instigation of doctors in certain districts.

After a long discussion the resolution was carried by twenty-six votes to nine.

### General Hospital, Birmingham.

PRESENTATION TO SIR THOMAS CHAVASSE.

On Thursday last week the Chairman of the Presentation Committee, Mr. J. B. Clarke, presented a portrait of Sir Thomas Chavasse, by Mr. Arthur T. Nowell, to be hung in the board room of the General Hospital, on behalf of 171 governors. The Lord Mayor, Lieutenant-Colonel Ernest Martineau (vice-president), accepted it gratefully on behalf of the hospital. Other presentations followed. Sir John C. Holder, the chairman of the house committee, presented a portrait of Sir Thomas Chavasse to Lady Chavasse, and Mr. Gilbert Barling, senior surgeon, asked the Lord Mayor to present an illuminated resolution and list of subscribers to Mr. Arthur Chavasse on behalf of his father, who was absent through having recently sustained an accident in the hunting-field.

The portrait of Sir Thomas for the General Hospital, which is three-quarter length, represents him standing facing the spectator nearly full face and wearing the gown of a Fellow of the Royal College of Surgeons. It was an apt recognition of his long connection with the hospital, for he was appointed assistant surgeon in 1877, and in 1881 began his work on the honorary staff, which he carried on for thirty-one years.

The address was illuminated by Messrs. Morton and Newey, on specially prepared vellum, and was in heraldic style of ornamentation and occupied seven pages. The lettering of the title-page was in gold letters, richly burnished, and a water-colour drawing of the new General Hospital formed the base of the border. On the second page the resolution was commenced and continued on the following page, followed by a list of 171 subscribers. A drawing of the old General Hospital was introduced, together with a sketch of operating theatre No. 1. The lettering on front cover: "To Sir Thomas Frederick Chavasse, F.R.C.S., Honorary Consulting Surgeon to the General Hospital, Birmingham, 1912." Then followed an extract from the Minutes of the Board of Management on Friday, March 1, 1912, which records the board's regret at Sir Thomas's resignation. The address concludes with the names of the 171 subscribers to the special fund.

### Buildings Contemplated and in Progress.

ARBROATH.—Erection of the new infirmary is about to be commenced; it is estimated to cost £14,500, and tenders for the several trades have been accepted.

AYR.—The Town Council has approved a scheme for a new hospital for phthisis patients estimated to cost £1,200.

BROMLEY.—The Bromley and Beckenham Joint Hospital Board has approved plans and specifications for additions and alterations at the isolation hospital.

CLAPHAM COMMON.—An anonymous gift of £25,000 has been made in aid of the proposed Women's Hospital at Clapham Common.

COATBRIDGE.—Additions are projected at the fever hospital estimated to cost £1,540, and a tender of £1,165 for a phthisis hospital has been accepted.

COLINTON.—The Public Health Committee recommend the Edinburgh Council to provide 100 additional beds for phthisis patients at Colinton Mains Hospital at a cost of about £5,000.

DARLINGTON.—It is proposed to erect a new ward for lunatics at the Guardians' Infirmary, and a site adjoining the infirmary on the east side has been selected.

DEVONPORT.—Mr. C. Cheverton, M.S.A., 64A Chapel Street, Devonport, is architect for a nurses' home at Ford Workhouse. Tenders are invited.

DEWSBURY.—Messrs. W. Hanstock and Son, Branch Road, Batley, are architects for alterations at Dewsbury Workhouse. Tenders are invited.

DURHAM.—Mr. H. T. Gradon, Market Place, Durham, is architect for works at Church Street Female Home for the Guardians. Tenders are invited.

EXETER.—The foundation-stone has been formally laid of the new home for children which is being erected by the Guardians from a design by Mr. R. M. Challice. The accepted tender was £6,359.

FALKIRK.—The Falkirk Council has selected a sanatorium site. The cost is estimated at £7,403.

HARROW.—The Local Government Board has held inquiry into the application of the Urban Council for sanction to a loan in connection with the isolation hospital.

HOWDEN.—An inquiry is to be held on February 5 into an application by Howden Rural Council for sanction to a £3,200 loan for the provision of an infectious diseases hospital.

ISLEWORTH.—Mr. N. Parr, M.S.A., architect to the Brentford Guardians, is responsible for proposed extensions to operating theatre, etc., at the infirmary. Tenders for the works are being considered.

KILMARNOCK.—New surgical wards and operating room, etc., are projected at Kilmarnock Infirmary. The cost is put at £14,000.

LICHFIELD.—It is proposed to instal a new hot-water supply and heating system at Lichfield Guardians' infirmary. The architect in charge is Mr. R. J. Barnes, of Bore Street, Lichfield, and tenders are now invited.

LONDON.—Mr. E. J. Harrison, 9 Gray's Inn Square, W.C., is architect for a new laundry at Tredegar Square, Mile End, E., for Mile End (Old Town) Guardians. The contract contains a fair-wages clause.

LONDON.—The Whitechapel Guardians have received ten tenders for a Lancashire boiler. The lowest is £334, the highest £415. The lowest tender has been sealed.

LONDON.—Lambeth Guardians have received eleven tenders for three electric lifts—two at the infirmary, Brook Street, and one at the workhouse, Renfrew Road. Messrs. Waygood's estimate, amounting to £1,566, to complete the work within fifteen weeks, has been accepted.

OUNDLE.—Oundle Urban Council is to seek sanction to a £1,200 loan for additional accommodation at the isolation hospital.

RUGBY.—The Guardians have adopted plans and estimates, amounting to £1,850, for a children's home.

RUGBY.—A new laundry is about to be erected at Rugby Workhouse. Plans have been approved at an estimated cost of £2,100.

ST. HELENS.—The Town Council has decided to obtain tenders for two pavilions and five double-bedded shelters at the proposed hospital at Eccleston Hall, and the Borough Surveyor is to prepare plans for other structural alterations.

SHEERNESS.—Alterations and additions are projected at the workhouse infirmary by the Guardians of Sheppey Union. Tender forms are to be obtained from the Master of the Workhouse, and are to be delivered not later than February 18.

SLIGO.—Mr. P. J. Kilgallen, borough surveyor, is architect for additions and alterations at the Guardians' premises The contract contains a fair-wages clause, and a surety bond is required.

SOUTHAMPTON.—The Corporation has decided to seek official sanction to a borrowing of £2,100 for structural improvements at the isolation hospital.

VICTORIA.—The Royal Jubilee Hospital, Victoria, B.C., is presently to be reconstructed.

WELLINGTON.—Mr. A. Jenkins, Market Street, Wellington, is architect for alterations at the workhouse for the Guardians of Wellington (Salop) Union. Sealed tenders are to be delivered not later than February 13, addressed to the Clerk, Mr. R. Gwynne, Edgbaston House, Wellington, Salop.

WILMINGTON.—Dartford Guardians have received authority from the Local Government Board to purchase Mount Pleasant Estate at a cost not exceeding £3,500 for a boys' home.

WIMBLEDON.—Inquiry into the Town Council's application for sanction to a £4,000 loan has been held. The loan is to provide a scarlet-fever convalescent pavilion at Gap Road Isolation Hospital.

WINCHMORE HILL.—Ten tenders have been received by the Metropolitan Asylums Board for two ironing machines at the Northern Hospital. Messrs. Bradford and Co.'s estimate of £376 12s. is recommended for acceptance.

### MANCHESTER AND DISTRICT ASSOCIATION OF HOSPITAL SECRETARIES.

The fifth annual dinner of the above Association was held on Saturday, January 25, 1913, at the Albion Hotel, Piccadilly, Manchester, under the presidency of Mr. Walter G. Carnt, F.C.I.S., the president. About forty members and their friends attended, and after the dinner a whist drive took place. This Association numbers amongst its members secretaries of voluntary hospitals in Lancashire, Cheshire, and Derbyshire, and was founded in 1908.

### Appointments.

Mr. F. Morres has been promoted to be third assistant medical officer at Cane Hill Mental Hospital.

Mr. John Macarthur, third assistant at Colney Hatch Mental Hospital, has been promoted to be second assistant medical officer.

Mr. S. Wyard has resigned the post of fourth assistant medical officer at Hanwell Mental Hospital, and has been succeeded by Mr. G. W. B. James.

Mr. C. F. Schuler has been appointed sixth assistant medical officer at Banstead Mental Hospital, in succession to Mr. M. J. McGrath, who has resigned.

Mr. J. Gray Clegg, M.B., F.R.C.S., has been appointed hon. ophthalmic surgeon to the Manchester Children's Hospital, and Miss Marion Stocks, M.B., has been appointed second assistant medical officer at the outpatients' department.

The following appointments have been made at the Royal Southern Hospital, Liverpool, viz., Mr. Arthur John Wallace, M.D., C.M., honorary consulting gynæcologist, and Mr. Herbert Williams, M.B., Ch.B., F.R.C.S. (Edin.), D.P.H. (Oxon.), honorary assistant surgeon.

### Colonial Appointments.

The King has been pleased to give directions for the appointment of Mr. James Cran, M.D., and Mr. Joseph Alan Dredge to be unofficial members of the Legislative Council of the Colony of British Honduras. Mr. Cran graduated M.B., M.S., at Aberdeen University in 1895.

### FOREIGN DECORATIONS.

The King has granted authority to accept and wear the Insignia of the Third Class of the Medjidieh, conferred upon him by the Khedive, authorised by the Sultan, in recognition of valuable services rendered by him, to Mr. Ll. P. Phillips, M.A., M.D., B.C., F.R.C.S., Professor of Medicine at the Medical School, Cairo.

### Obituary.

Dr. William Carter, for many years Professor of Materia Medica and Therapeutics at Liverpool University, has died, aged seventy-six. He was an active promoter of the Liverpool School of Tropical Medicine, and had charge of the Tropical Diseases Ward at the hospital. He had strong views on other subjects besides the importance of tropical medicine, however, for he strongly advocated total abstinence from alcohol and tobacco.

### Gifts and Bequests.

Mr. James A. Reid, LL.D., has bequeathed, subject to the life interest of his sister, £500 to the Royal Hospital for Sick Children, Glasgow, to name a cot in memory of his wife.

Mr. George McGregor, of Dundee, retired hose pipe manufacturer, has bequeathed £250 each to the Royal Victoria Hospital, Dundee, the Dundee Orphan Institute, and the Bannatyne Home of Rest, Newtyle.

Mr. Mark Kippax, of Blackpool, retired cotton manufacturer, subject to one life interest, has bequeathed £1,000 each to the Victoria Hospital for Burnley and district and to the Victoria Hospital, Blackpool.

Mrs. Sarah Annie Ward, of Askham Bryan Vicarage, York, who left estate valued at £53,093 gross, has bequeathed £4,000 to the Cancer Hospital at Brompton and £150 to the Men's Convalescent Home, Rhyl.

Messrs. John Summers and Sons, of Hawarden Bridge Ironworks, are contributing £3,000 towards the Chester Infirmary Extension Fund. The infirmary is being enlarged and renovated as a memorial to King Edward.

Mr. William Townsend White, of Cardiff, retired butcher, has bequeathed £500 to King Edward VII.'s Hospital, Cardiff; £500 to the Bridgwater Infirmary; £300 to the Royal Hamadryad Seamen's Hospital, Cardiff Docks; and £200 to the Institution for the Blind, Cardiff.

### Personalia.

Sir Robert W. Philip, M.D., has been elected vice-chairman of the National Association for the Prevention of Consumption, in succession to the late Dr. Theodore Williams.

Dr. Hughes, the resident house surgeon at the Bradford Royal Infirmary, has resigned his position and has gone to Lytham, where he has suddenly been attacked with appendicitis, from which, however, he is now recovering.

Miss Melville, of Queen Margaret College, has been appointed convener of the Glasgow Women's Private Hospital, in succession to Mrs. D. M. Ross, who has retired. The honorary secretary is Miss Charlotte Bannerman, and Dr. Alice McLaren is senior medical officer.

### THE LONDON COUNTY AND WESTMINSTER BANK.

Little better evidence of the prosperity of the London County and Westminster Bank can be found than in the net profits which were declared at the annual meeting last week as £1,055,479, with a dividend for the year of $21\frac{1}{4}$ per cent., less tax. £200,000 has been appropriated for writing down investments, which, as the Chairman explained, must not be regarded as money lost, but as a sort of special reserve which is earning interest for the benefit of the shareholders. The figures of the balance sheet are given in detail in our business columns, to which readers are referred.

